# Comparative Investigation of the Chemiluminescent Properties of a Dibrominated Coelenterazine Analog

**DOI:** 10.3390/ijms23158490

**Published:** 2022-07-30

**Authors:** João Sousa, Carla M. Magalhães, Patricia González-Berdullas, Joaquim C. G. Esteves da Silva, Luís Pinto da Silva

**Affiliations:** 1 Centro de Investigação em Química (CIQUP), Instituto de Ciências Moleculares (IMS), Departamento de Geociências, Ambiente e Ordenamento do Território, Faculdade de Ciências, Universidade do Porto, Rua do Campo Alegre s/n, 4169-007 Porto, Portugal; up201904589@edu.fc.up.pt (J.S.); up201201533@edu.fc.up.pt (C.M.M.); patricia.berdullas@fc.up.pt (P.G.-B.); jcsilva@fc.up.pt (J.C.G.E.d.S.); 2 LACOMEPHI, GreenUPorto, Departamento de Geociências, Ambiente e Ordenamento do Território, Faculdade de Ciências, Universidade do Porto, Rua do Campo Alegre s/n, 4169-007 Porto, Portugal

**Keywords:** coelenterazine, chemiluminescence, bioluminescence, superoxide anion, luminescence, coelenterazine analogues, *Cypridina* luciferin

## Abstract

Chemi- and bioluminescence are remarkable light-emitting phenomena, in which thermal energy is converted into excitation energy due to a (bio)chemical reaction. Among a wide variety of chemi-/bioluminescent systems, one of the most well-known and studied systems is that of marine imidazopyrazinones, such as Coelenterazine and *Cypridina* luciferin. Due to the increasing usefulness of their chemi-/bioluminescent reactions in terms of imaging and sensing applications, among others, significant effort has been made over the years by researchers to develop new derivatives with enhanced properties. Herein, we report the synthesis and chemiluminescent characterization of a novel dibrominated Coelenterazine analog. This novel compound consistently showed superior luminescence, in terms of total light output and emission lifetime, to natural imidazopyrazinones and commercially available analogs in aprotic media, while being capable of yellow light emission. Finally, this new compound showed enhanced chemiluminescence in an aqueous solution when triggered by superoxide anion, showing potential to be used as a basis for optimized probes for reactive oxygen species. In conclusion, bromination of the imidazopyrazinone scaffold appears to be a suitable strategy for obtaining Coelenterazines with enhanced properties.

## 1. Introduction

Chemiluminescence (CL) and bioluminescence (BL) are light-emitting phenomena that consist of the conversion of thermal energy into excitation energy through a chemical reaction, with BL being a subtype of CL that occurs in living organisms, such as jellyfish, bacteria, fish, etc., and that is catalyzed by an enzyme [1,2]. CL/BL reactions typically involve the formation of a high-energy intermediate, based on a peroxide, which decomposes releasing its energy and allowing the direct chemiexcitation to singlet excited states [3,4,5].

CL and BL reactions/systems present amazing properties that are relevant in different fields, such as analytical chemistry, molecular biology, and medicine since they have high quantum yields, a high signal-to-noise ratio and are nontoxic, which allows a noninvasive and in real-time sensing and imaging of targeted molecules or processes, both in vitro and in vivo [5,6,7]. The fact that these systems are independent of an external light source for activation also eliminates the problem of light penetration into biologic tissues, except for the resulting light emission. Besides its applications in real-time imaging [6,8,9], these systems show anticancer activity [10,11,12] but are mostly used in sensing applications [13,14,15].

**Scheme 1:**
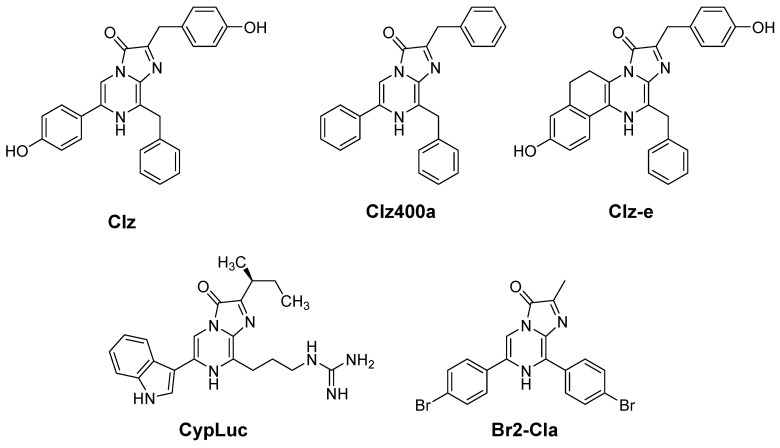
Molecular structures of Clz, Clz400a, Clz-e, CypLuc and Br2-Cla.

One of the most studied CL/BL substrates is Coelenterazine (Clz, Scheme 1) [1,3,16,17,18,19,20,21], which is present in many marine BL organisms [22]. Clz is capable of both BL (with luciferase enzymes or photoproteins) [3] and CL (triggered without an enzyme/protein by oxygenation) [23,24,25]. These Clz-based reactions follow a mechanism that consists in two steps: first, there is the oxygenation of the imidazopyrazinone core that leads to the formation of dioxetanone, the high-energy peroxide intermediate; the dioxetanone will undergo thermolysis almost instantly due to its high instability, during which the reacting molecules can cross directly to singlet excited states, resulting in the chemiexcited light emitter coelenteramide [1,3,4,20].

Another example of natural CL/BL imidazopyrazinone compounds found in marine species is *Cypridina* luciferin (CypLuc, Scheme 1), which can be found in the ostracod *Cypridina hilgendorfii* [23,24]. Besides natural imidazopyrazinones, synthetic analogs have also been developed over the years, with some being already commercialized. Two of the most well-known examples are Coelenterazine 400a (Clz400a, Scheme 1) and Coelenterazine-e (Clz-e, Scheme 1). Clz400a generates light with an emission maximum at ~400 nm, and it is generally used for bioluminescence resonance energy transfer (BRET) in the presence of *Renilla* luciferase due its minimal interference with the emission of fluorescent acceptors [25,26]. As for Clz-e, it possesses an additional ethyl group to Clz, which forms an additional ring system. Moreover, it is generally associated with enhanced emission when compared with Clz [5,27].

It should be noted that besides the already commercialized Clz-based analogs (such as Clz400a and Clz-e), there has been made a significant effort by the research community to develop new Clz-based molecules with enhanced properties, such as brighter light-emission, red-shifted emission, and longer emission half-life [19,21,25,26,28,29,30,31,32,33]. This results from the increasing usefulness of both the BL and CL reactions of Clz and related imidazopyrazinones, in different fields, such as bioimaging [6,34,35,36] and sensing [33,37,38]. 

In more recent years, our group has also been active in the development of novel Clz analogs with enhanced properties, with a focus on the introduction of single bromine heteroatoms into the imidazopyrazinone scaffold [2,10,11,12]. More specifically, some monobrominated Clz analogs (Cla compounds) showed tumor-selective anticancer activity, while showing a relevant profile of safety [10,11,12]. Another monobrominated Cla compound showed significantly enhanced CL in an aqueous solution when compared with Clz (intensities 2.13 × 10^1^ to 1.11 × 10^4^ times higher) at different pH conditions [2]. Following on this experience, we report now for the first time the development of a dibrominated Cla compound, Br2-Ca (Scheme 1). For its development, both the phenol and benzyl groups of Clz were replaced by a bromophenyl moiety, while the *p*-cresol moiety was replaced by a methyl group (Scheme 1). Its CL reaction was investigated in both aprotic media and aqueous solution while comparing it with that of Clz, CypLuc, Clz400a and Clz-e. In aprotic media, Br2-Cla was found to possess brighter light-emission than Clz and CypLuc, and comparable emission to Clz400a, while possessing lower emission than Clz-e. More relevantly, while typical Clz-based molecules emit blue CL light, Br2-Cla was found to emit yellow CL light, which is a significant, red-shifted emission. Finally, Br2-Cla was also found to possess enhanced emission in an aqueous solution. Thus, this new dibrominated Cla compound was found to be superior as a CL-emitting agent than Clz itself, as well as other natural and synthetic/commercial Clz-based molecules.

## 2. Results and Discussion

### 2.1. Chemistry and Spectroscopic Properties of Br2-Cla

Br2-Cla was synthesized by following previous methodologies already validated and optimized by the present team [2,10,11,12]. The procedures are described in more detail in the Supporting Information, but (in short) consist of an initial Suzuki–Miyaura cross-coupling between commercial 3,5-dibromopyrazin-2-amine and (4-bromophenyl)boronic acid. This results in the formation of aminopyrazine derivatives disubstituted with 4-bromophenyl moieties. The second and last step consisted of a cyclization reaction between this intermediate with methylglyoxal to afford Br2-Cla. The structure of this latter compound and of the synthesis intermediates was confirmed with both ^1^H/^13^C NMR (Figures S1–S3) and FT-MS spectroscopy (Figure S4).

**Figure 1:**
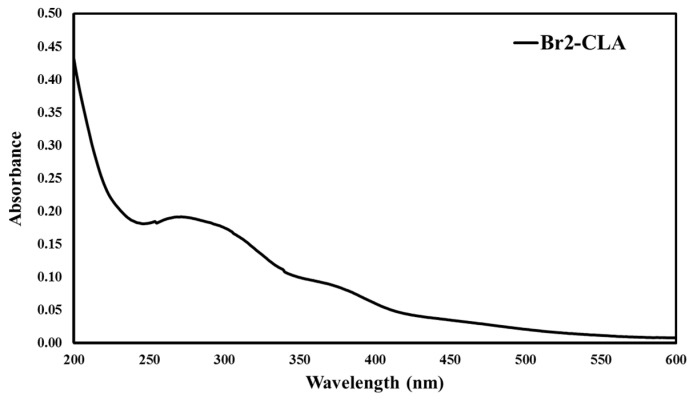

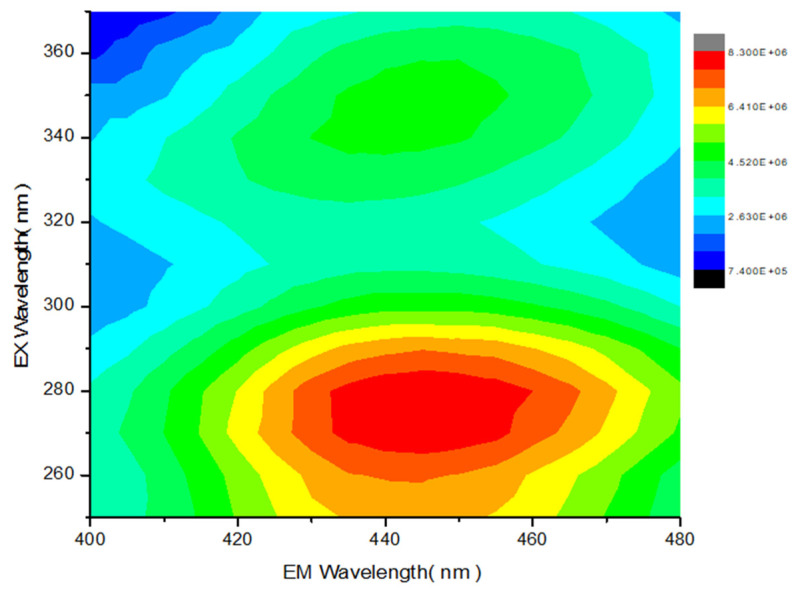
UV-Vis (**top**) and 2D EEM contour plot (**bottom**) for Br2-Cla (20 μM) in aqueous solution.

Subsequently, we proceeded to further characterize Br2-Cla, which we started by determining its spectroscopic properties. Namely, we started by measuring its UV-Vis spectrum in aqueous solution (Figure 1). This spectrum shows sharp absorption between 200 and 250 nm, as well as a more well-resolved band at ~275 nm. A not-so-well-defined band is also found at ~370 nm. A small shoulder can be observed at about 450 nm. This type of UV-Vis spectra is similar to what was found for other brominated Cla compounds developed by our group [2,10,11,12].

The fluorescence of Br2-Cla in an aqueous solution was also measured, with the corresponding 2D excitation-emission matrix (EEM) contour plot being presented also in Figure 1. The EEM plot shows the presence of two emissive centers with the same fluorescence wavelength maxima (~445 nm), but with two excitation wavelength maxima (~275 and ~355 nm). Interestingly, the light intensity is higher at the emissive center excited by lower wavelengths. As there is an overlap between excitation and absorption bands, the two emissive centers are attributed to the excitation of different singlet excited states, which are converted into the same lowest emissive singlet excited state (in accordance with Kasha’s rule) [39].

### 2.2. Investigation of Chemiluminescence in Aprotic Solvents

The next step of this study was then to characterize the CL properties of Br2-Cla in aprotic solvents. More specifically, in DMF and DMSO. It is well known that imidazopyrazinones are able to spontaneously emit CL when added to these solvents, without the need for any catalyst, while emitting stable signals [2,5,23,30,31]. Furthermore, the CL reaction mechanism in aprotic solvents of Clz and derivatives is similar to that of BL [30,31,40,41], which indicates that the study of the former allows some insight into the latter without the need for working with enzymes, which are particularly expensive, complex and time-consuming.

**Figure 2:**
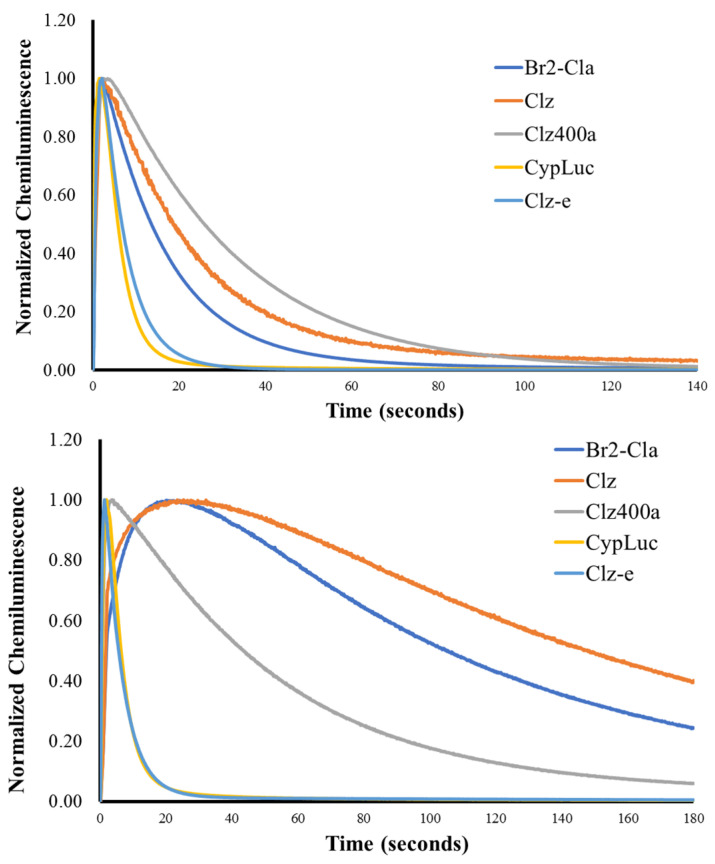
Normalized CL emission as a function of time (in seconds) for Br2-Cla, Clz, Clz400a, CypLuc and Clz-e, in DMSO (**top**). Normalized CL emission as a function of time (in seconds) for Br2-Cla, Clz, Clz400a, CypLuc and Clz-e, in DMF (**bottom**). All assays were performed with a concentration of 0.73 µM for all compounds.

Given this, we have measured the CL kinetic profiles of Br2-Cla in both DMF and DMSO, which were compared with those obtained for Clz, CypLuc, Clz400a and Clz-e in the same conditions. By performing this comparison, it is easier to evaluate the usefulness of the CL properties of the novel dibrominated Cla compound. The normalized CL kinetic profiles are presented in Figure 2.

We can see in Figure 2 that all reactions that took place in DMSO have a similar flash profile, with an immediate burst of light followed by decay to basal levels (in a tens of seconds timescale). Thus, the CL kinetic profile of Br2-Cla is qualitatively identical to that of the natural Clz/CypLuc and synthetic/commercial Clz400a and Clz-e. However, by visual inspection, it is already clear that the emission lifetime of Br2-Cla is longer than that of CypLuc and Clz-e, while being shorter than Clz400a and Clz. As for CL reactions that took place in DMF (Figure 2), CypLuc and Clz-e appear to possess qualitatively identical profiles to those found in DMSO. However, the emission lifetimes for Br2-Cla, Clz400a and Clz appear to be relevantly longer in DMF than in DMSO.

Quantitative analysis was subsequently performed by measuring the light-emission intensity maximum (E_max_, in relative lights unit, RLU), calculated area of total light-emission (area, in RLU), the CL half-life values (t_half_, in s), and the initial velocities of the CL reaction (v_initial_, in RLU s^−1^) for the CL reactions of all compounds in both DMSO (Table 1) and DMF (Table 2) [2,5,42,43].

By analysis of Table 1, we can conclude that in DMSO, Br2-Cla presents a relatively bright CL emission. Namely, its E_max_ is significantly higher than Clz, Clz400a and CypLuc. In terms of total light output (area), the emitted CL of Br2-Cla is significantly higher than both Clz and CypLuc, while being comparable with that of Clz400a. In fact, in terms of light-emission, Br2-Cla is only second to Clz-e. Nevertheless, the difference between both compounds is significant, which is not unexpected as Clz-e is known for its enhanced emission regarding Clz [5]. Given this, Br2-Cla is found to be quite superior in CL emission intensity to both natural Clz and CypLuc. In terms of v_nitial_, Br2-Cla also presents significantly higher values than Clz, Clz400a and CypLuc, and only behind Clz-e, which indicates that its CL reaction in DMSO should be quite efficient (when compared with other Clz-based molecules). Interestingly, Br2-Cla presents somewhat intermediate t_half_ values, as the emission lifetime of this compound is relatively higher than CypLuc and Clz-e, while being lower than both Clz and Clz400a. It should be noted that higher t_half_ are beneficial for practical applications of CL/BL, as higher values facilitate the measurement of the analytical signal. Thus, the fact that the t_half_ of Br2-Cla is about double that presented by Clz-e is an advantage of the former compound, despite higher CL intensities emitted by the latter.

Table 2 presents the E_max_, area, t_half_ and v_initial_ values for all studied compounds in DMF. Br2-Cla still presents higher light-intensity maxima than both Clz and Clz400a. However, in this solvent, CypLuc presents now significantly higher E_max_ values than Br2-Cla. It should be noted that previous studies have already reported higher CL emission of CypLuc in DMF than in DMSO [5], which could explain this variation. Clz-e is still the molecule with higher emission intensity maxima. As for total light output, Br2-Cla presents brighter CL than both Clz and Clz400a. Interestingly, while the area values for CypLuc and Clz-e are higher than for Br2-Cla, these differences are not so significant (especially for CypLuc). This indicates that the higher E_max_ of the former should result from a more intense and quick initial burst of light, and not from higher total emission. In fact, we can see that while the v_initial_ values are larger for CypLuc/Clz-e than for Br2-Cla, the situation is clearly reversed in terms of t_half_. In fact, the emission lifetime of Br2-Cla is about 10 times higher than for CypLuc/Clz-e. Given this, it can be concluded that in DMF, Br2-Cla possesses superior CL to both Clz and Clz400a, while presenting comparable light emission to that of CypLuc and Clz-e.

**Figure 3:**
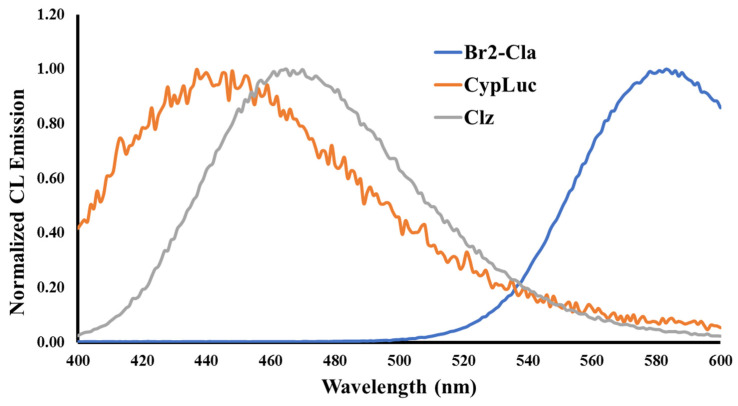
Normalized chemiluminescent emission spectrum of Br2-Cla, CypLuc and Clz, measured in DMF.

While natural Clz-based species (as Clz itself and CypLuc) typically emit blue light during their CL/BL reactions [5,23], a more red-shifted emission would be more suitable for biological applications to reduce light-absorption by tissues [44]. So, we also evaluated the CL spectrum of Br2-Cla in an aprotic medium, while comparing it with the spectra of natural Clz and CypLuc (Figure 3). As expected, [40,45], the two natural imidazopyrazinone molecules presented blue emission, with Clz emitting light with a wavelength maximum of ~470 nm, while CypLuc presented an emission wavelength maximum of ~430 nm. As for Br2-Cla, it emits yellow light with an emission wavelength maximum at ~580 nm, which is significantly red-shifted (by ~110–150 nm) when compared with natural Clz/CypLuc. Thus, besides having generally brighter CL emission than the natural compounds (especially Clz), Br2-Cla also generates light with color more suitable for practical applications in biological media [44]. 

After comparing the CL reaction of Br2-Cla with that of natural and synthetic/commercial Clz-based compounds, we proceeded to analyze the potential effect of pH in its CL reaction in aprotic media. To that end, we have measured the CL kinetic profile of Br2-Cla in DMSO and DMF in both acidic and basic media, with results being presented in Table 3. Measurements in acidic media were performing by the addition of sodium acetate buffer pH 5.2 (1%), and in basic media by the addition of NaOH (0.1 M), which are typical conditions used in previous CL-based studies [2,5,30,31,40,41,45]. It is clear by analysis of Table 3 that pH has a significant effect on the CL reaction of Br2-Cla, which is similar in both solvents. That is, the light-emission maxima (E_max_) are significantly higher in basic media. However, the total light output (area) is identical at both pH conditions in DMF, while being slightly higher at acidic media in DMSO. This can be rationalized by analyzing the values of t_half_ and v_initial_. Namely, the first decreases significantly with pH, while the second has the opposite relationship. This indicates that the kinetics of the CL reaction of Br2-Cla are significantly quicker in basic pH than in acidic media, which results in an “explosive” burst of light that decays almost instantly to more basal levels. On the other hand, in acidic pH, the reaction occurs more slowly and without an initial burst of light and subsequent decay to basal levels. In fact, it appears that while the light is emitted during a longer period of time at acidic media, the same (or even higher) total light output is produced at different pH conditions. Thus, the pH appears to affect the kinetics of the CL reaction of Br2-Cla, but not the total light output.

The pH-dependency of the CL reaction of imidazopyrazinones is well-known [2,30,31,40,41,45], with one explanation for it being the chemical equilibria of dioxetanone, the high-energy intermediate which thermolysis allows for chemiexcitation [1,3,4,20]. Namely, it could be expected that dioxetanone is in a neutral state at acidic pH, while being deprotonated at basic media [4,20,46]. More importantly, the thermolysis of neutral dioxetanone is typically associated with significantly higher activation energies than that of its corresponding anionic species [47,48,49,50]. These differences can help to explain the pH effect on the kinetics of Br2-Cla (Table 3). There is also some evidence that indicates that neutral dioxetanones could lead to more efficient chemiexcitation than anionic species [31,40,45,51], which is in line with higher total output measured in DMSO at acidic pH.

### 2.3. Investigation of Chemiluminescence in Protic Solvents

Besides generating CL in aprotic media, Clz-based compounds are also known to emit light in an aqueous solution when triggered by superoxide anion [52]. More specifically, the superoxide anion can act as a trigger of the CL reaction, by inducing the oxygenation step, which results in the dioxetanone intermediate (from which results in the chemiexcited light-emitter) [3,23,52]. This is an attractive feature for this type of molecule, given that the superoxide anion is involved in intra-/intercellular signaling pathways [53], and its unbalance can lead to deleterious health conditions, such as inflammation, cancer and chronic granulomatous disease [54,55]. Thus, the research community has been performing a relevant effort in the development and validation of Clz and analogs as CL-based probes for the sensitive and dynamic sensing of superoxide anion [2,23,37,38].

Given this, the next step of our study was to determine if Br2-Cla could generate CL in an aqueous solution when triggered by superoxide anion. To that end, the CL of this compound was obtained with a luminometric approach, in the presence of increasing amounts of potassium superoxide (5, 10 and 15 mg). This latter compound is a source of superoxide anion in protic solvents and used before in the study of Clz/Cla-based CL reactions [2,10,12]. It should be noted that superoxide anion can be quite unstable in protic media, especially in aqueous solution, due to strong solvation and spontaneous disproportionation [56,57]. Given this instability, we only measured the initial light-emission intensity maxima (E_max_), which are presented in Table 4. For comparison purposes, we have also measured the superoxide anion-triggered CL emission of both Clz and Clz-e (Table 4).

By analysis of Table 4, it can be seen that Br2-Cla does emit light when triggered by superoxide anion. Interestingly, it can be seen that increasing the amount of superoxide anion decreases the E_max_ value, meaning that this CL system is sensitive to the amounts of this ROS species. However, this decrease in light-emission with increasing amounts of superoxide anion does indicate that this oxidant can lead to over-oxidation of Br2-Cla. That is, increasing amounts of this ROS species does not only lead to triggering the oxygenation step of the CL reaction but also leads to excessive oxidation of the CL substrate (and its possible degradation). Nevertheless, as the CL reaction of Br2-Cla is still sensitive to the amount of superoxide anion, obtaining relationships between analytical signal and analyte concentration is still possible. It should be noted that this relationship between superoxide anion amount and emitted CL was also found for a monobrominated Cla compound, also recently developed by us [2]. This indicates that this tendency to over-oxidation by superoxide anion could be related to the bromination of the imidazopyrazinone core. In fact, analysis of Table 4 indicates that natural Clz should be more resistant to over-oxidation, as its E_max_ values increase with an increasing amount of superoxide anion, as expected from the literature [2,37].

More relevantly, we can see in Table 4 that, once again (as in Table 1 and Table 2), Br2-Cla presents significantly enhanced CL emission when compared with Clz. In fact, the emission of Br2-Cla at higher amounts of superoxide anion (~56%), when it is decreased, it is still significantly higher than that of Clz (~8%), in conditions in which it is increased. Furthermore, and contrary to what was seen in DMF and DMSO, the CL emission intensity of Br2-Cla is even significantly higher than that of Clz-e, which is known to be a commercial Clz analog with enhanced emission.

This emission enhancement of Br2-Cla is particularly relevant if we think that CL reactions tend to possess lower light-emitting intensities in aqueous solution (due to factors, such as energy loss to water molecules) [58,59], with Clz not being an exception [60]. Thus, the enhanced emission of Br2-Cla indicates that bromination of Clz is a solution to this problem. In fact, the monobrominated Cla compound developed by us recently [2], and already mentioned, also presented enhanced emission in an aqueous solution when compared with Clz, which supports the notion that bromination of the imidazopyrazinone scaffold is responsible for the higher emission intensities in aqueous solutions. It should be noted, however, that the monobrominated derivative emitted blue light (~460 nm) [2], similarly to Clz, while Br2-Cla emitted yellow light (~580 nm). Thus, the latter compound appears to possess some advantages, in terms of emission color, for potential applications with biological relevance in the future.

Given these results, Br2-Cla shows significantly enhanced superoxide anion-triggered CL emission when compared with an already validated CL probe for this ROS species (Clz), which indicates that Br2-Cla shows potential for future sensing applications.

Finally, it should be noted that the enhanced emission of Br2-Cla is also quite interesting because it is observed, despite the presence of two bromine heteroatoms in its structure. More specifically, bromine heteroatoms typically lead to fluorescence quenching due to the heavy-atom effect (which enhances intersystem crossing to triplet states). However, here the bromine heteroatoms appear to have an opposite effect. The absence of a quenching effect in Br2-Cla is also in line with the enhanced emission in an aqueous solution of monobrominated derivatives developed recently by us [2]. Thus, our results indicate that specific additions to bromine heteroatoms in Clz-based structures can lead to an enhancement effect, which should be further explored in the future.

## 3. Materials and Methods

### 3.1. Synthesis of Br2-Cla

The chemical synthesis of Br2-Cla is described in detail in the Supporting Information. In summary, the initial step of the synthesis consisted of a Suzuki–Miyaura cross-coupling reaction between commercial 3,5-dibromopyrazin-2-amine and (4-bromophenyl)boronic acid in THF, which yielded the dibrominated intermediate 3,5-bis(4-bromophenyl)pyrazin-2-amine. The second and last step consisted of the formation of the imidazopyrazinone core by reacting the synthesis intermediate with methylglyoxal in acidic media, to afford Br2-Cla. The structural characterization of the synthesis intermediate and Br2-Cla was performed by ^1^H/^13^C-NMR spectroscopy (Figures S1–S3) and FT-MS spectrometry (Figure S4).

### 3.2. Clz, Clz400a, Clz-e and CypLuc

Clz, Clz400a, Clz-e and CypLuc were all purchased from NanoLight Technology and were dissolved in methanol and stored at −20 °C.

### 3.3. Luminometric and Spectroscopy Characterization

Measurements of UV-Vis spectra were accomplished using a VWR^®^ UV-Vis Spectrophotometer (UV-3100PC) with a quartz cuvette, at room temperature. A Horiba Jovin Fluoromax 4 spectrofluorometer was used to measure the fluorescence spectra using an integration time of 0.1 s. Slit widths of 5 nm were used for both the excitation and emission monochromators. The chemiluminescent spectra were measured with the same spectrofluorometer, but with a slit width of 29 nm for the emission monochromators. Quartz cuvettes were employed for both the chemiluminescent and fluorescent spectra. The chemiluminescent kinetics were measured in a homemade luminometer, in which a Hamamatsu HC135-01 photomultiplier tube is found. 

The chemiluminescent reactions took place at room temperature and were all performed at least in sextuplicate. The light was integrated and recorded in 0.1 s intervals, while typical assays were measured for 4 min after the initial burst of light. Measurements were performed in both aprotic and protic solvents. The considered aprotic solvents were dimethyl sulfoxide (DMSO) and *N*,*N*-dimethylformamide (DMF), while water was chosen as the protic solvent. Measurements at different pH were also performed by the addition to DMSO/DMF of either sodium acetate buffer pH 5.2 (1%) or NaOH (0.1 M). The chemiluminescent reactions took place in protic solvent (water) by the addition of increasing amounts of potassium superoxide (5, 10 and 15 mg). Assays were performed with concentrations of the chemiluminescent compounds of 0.73 μM and 3 μM in aprotic and protic solvents (respectively).

## 4. Conclusions

It was reported for the first time the development of a novel dibrominated Clz analog, Br2-Cla, which CL reaction was investigated in different aprotic and protic media in comparison to both natural Clz/CypLuc and commercially-available analogs Clz400a/Clz-e. In aprotic media, Br2-Cla showed superior CL (in terms of light output) to both Clz400a and Clz. The novel analog also showed brighter luminescence than CypLuc in DMSO, while both compounds showed similar light-emission output in DMF. Interestingly, while Clz-e showed brighter luminescence than Br2-Cla in both aprotic media, the latter compound showed a significantly higher emission lifetime (which is an attractive feature for the better detection of the analytical signal). Furthermore, while Clz-based compounds typically emit blue light, Br2-Cla showed a relevantly red-shifted emission, by emitting yellow light (~580 nm). The CL reaction of Br2-Cla is also pH-dependent in aprotic media, especially in terms of its kinetics. That is, the reaction is significantly quicker in basic media than at acidic pH. Finally, Br2-Cla is also capable of CL in an aqueous solution, when triggered by superoxide anion, a ROS species of biological interest. More importantly, the superoxide anion-based CL reaction of this dibrominated analog was significantly enhanced when in comparison with natural Clz and commercially-available analogs. This indicates that Br2-Cla could be used as a basis for the development of optimized CL-based probes for the dynamic and sensitive sensing of superoxide anion. In short, the present results indicate that the bromination of the imidazopyrazinone core is a suitable approach for the development of novel Clz-based compounds with enhanced CL/BL emission.

## 5. Patents

WO20219211808—Chemiluminescent Imidazopyrazinone-Based Photosensitizers with Available Singlet and Triplet Excited States.

## Figures and Tables

**Table 1:** Light-emission intensity maximum (E_max_, in RLU), calculated area of total light-emission (area, in RLU), light-emission half-life (t_half_, in s) and initial velocities (v_initial_, in RLU s^−1^), for CL reactions of Br2-Cla, Clz, Clz400a, CypLuc and Clz-e in DMSO. Measurements were performed in solutions with a final concentration of 0.73 μM. Values are presented in percentage (%), with the results for Br2-Cla as reference.

Molecules	E_max_	Area	t_half_	v_initial_
Br2-Cla	100 ± 10%	100 ± 10%	14 ± 1 s	100 ± 11%
Clz	33 ± 6%	14 ± 2%	19.2 ± 2.2 s	55 ± 11%
Clz400a	61 ± 2%	103 ± 5%	24.8 ± 0.5 s	66 ± 4%
CypLuc	40 ± 3%	15 ± 1%	5.9 ± 0.1 s	55 ± 6%
Clz-e	696 ± 45%	272 ± 17%	6.1 ± 0.3 s	702 ± 84%

**Table 2:** Light-emission intensity maximum (E_max_, in RLU), calculated area of total light-emission (area, in RLU), light-emission half-life (t_half_, in s) and initial velocities (v_initial_, in RLU s^−1^), for CL reactions of Br2-Cla, Clz, Clz400a, CypLuc and Clz-e in DMF. Measurements were performed in solutions with a final concentration of 0.73 μM. Values are presented in percentage (%), with the results for Br2-Cla as reference.

Molecules	E_max_	Area	t_half_	v_initial_
Br2-Cla	100 ± 4%	100 ± 8%	79.8 ± 10.2 s	100 ± 10%
Clz	54 ± 8%	81 ± 11%	164.0 ± 14.4 s	51 ± 11%
Clz400a	69 ± 8%	64 ± 14%	73.7 ± 17.6 s	90 ± 13%
CypLuc	413 ± 37%	115 ± 5%	9.5 ± 1.2 s	317 ± 51%
Clz-e	587 ± 71%	156 ± 17%	8.2 ± 1.3 s	676 ± 126%

**Table 3:** Light-emission maximum (E_max_, in RLU), calculated area of light-emission (area, in RLU), light-emission half-life (t_half_, in s) and initial velocities (v_initial_, in RLU s^−1^), for the CL reactions of Br2-Cla in DMSO and DMF, at either at basic (NaOH, 0.1 M) or acidic pH (sodium acetate buffer pH 5.2, 1%). Measurements were performed in solutions with a final concentration of the compounds of 0.73 µM. Values are presented in percentage (%), with the results obtained in the presence of sodium acetate buffer pH 5.12 (1%) being the reference for both solvents.

Solvent	E_max_	Area	t_half_	v_initial_
DMSO—sodium acetate buffer pH 5.2 (1%)	100 ± 10%	100 ± 4%	40.0 ± 4.8 s	100 ± 24%
DMSO—NaOH (0.1 M)	222 ± 11%	88 ± 4%	0.48 ± 0.02 s	1517 ± 30%
DMF—sodium acetate buffer pH 5.2 (1%)	100 ± 4%	100 ± 5%	108.5 ± 5.4 s	100 ± 19%
DMF—NaOH (0.1 M)	1253 ± 105%	96 ± 4%	0.48 ± 0.05 s	1999 ± 252%

**Table 4:** Light-emission maximum (E_max_, in RLU) for the CL reactions of Br2-Cla, Clz and Clz-e at different concentrations of potassium superoxide (KO_2_) (5–15 mg). Measurements were performed in aqueous solutions with a final concentration of the compounds of 3 µM. Values are presented in percentage (%), with the results for Br2-Cla—10 mg as reference.

Molecule—KO_2_ Amount	E_max_
Br2-Cla—5 mg	109 ± 9%
Br2-Cla—10 mg	100 ± 8%
Br2-Cla—15 mg	56 ± 7%
Clz—5 mg	3 ± 1%
Clz—10 mg	6 ± 1%
Clz—15 mg	8 ± 1%
Clz-e—10 mg	35 ± 7%

## Data Availability

Data is contained within the article or Supplementary Material.

## Supplementary Materials

The following supporting information can be downloaded at: https://www.mdpi.com/article/10.3390/ijms23158490/s1.
