# Supplementary material for: Comparative Investigation of the Chemiluminescent Properties of a Dibrominated Coelenterazine Analog
Source: Int J Mol Sci. 2022 Jul 30;23(15):8490. doi: 10.3390/ijms23158490 (PMC9369366; doi:10.3390/ijms23158490)
Supplement: Supplementary file 1 [file ijms-23-08490-s001.zip › ijms-1832584-supplementary.pdf]

Supporting Information for

Comparative Investigation of the Chemiluminescent Properties of a Dibrominated Coelenterazine Analog

João Sousa,[1] Carla M. Magalhães,[1] Patricia González-Berdullas,[1] Joaquim C.G. Esteves da Silva,[1,2] Luís Pinto da Silva[1,2,]*

[1] Centro de Investigação em Química (CIQUP), Instituto de Ciências Moleculares (IMS), Departamento de Geociências, Ambiente e Ordenamento do Território, Faculdade de Ciências, Universidade do Porto, Rua do Campo Alegre s/n, 4169-007 Porto, Portugal.

[2] LACOMEPHI, GreenUPorto, Departamento de Geociências, Ambiente e Ordenamento do Território, Faculdade de Ciências, Universidade do Porto, Rua do Campo Alegre s/n, 4169-007 Porto, Portugal.

* Corresponding author: luis.silva@fc.up.pt.

Table of Contents

1. Experimental section S3

1.1. General Procedures S3

1.2. Instruments and data analysis S3

1.2.1. NMR S3

1.2.2. FT-MS S4

1.3. Synthesis and characterization S4

1.3.1. 3,5-*bis*(4-Bromophenyl)pyrazin-2-amine S4

1.3.2. 6,8-*bis*(4-Bromophenyl)-2-methylimidazo[1,2-*a*]pyrazin-3(7*H*)-one S5

2. Supporting figures S6

Figure S1. ^{1}H-NMR spectrum of 3,5-bis(4-bromophenyl)pyrazin-2-amine in $CDCl_3$ S6

Figure S2. ^{13}C-NMR spectrum of 3,5-bis(4-bromophenyl)pyrazin-2-amine in $CDCl_3$ S6

Figure S3. ^{1}H-NMR spectrum of 6,8-bis(4-bromophenyl)-2-methylimidazo[1,2-*a*]pyrazin-3(7*H*)-one in Methanol-d_4 .... S7

Figure S4. FT-MS spectrum of 6,8-bis(4-bromophenyl)-2-methylimidazo[1,2-*a*]pyrazin-3(7*H*)-one S7

1. Experimental section

1.1. General Procedures

Reagents and solvents were purchased from Millipore Sigma (Merck KGaA, Darmstadt, Germany) and used without further purification. All reactions involving oxygen or moisture-sensitive compounds were carried out under dry nitrogen atmosphere. Ice-water and silicon baths were used for reactions at low and high temperatures, respectively, with all reaction temperatures referring to the external bath. Organic extracts were dried over anhydrous Na_2SO_4, filtered and concentrated using a rotary evaporator (Büchi® Rotavapor® R-210, Büchi® B-491 Heating Bath 120V, KNF Neuberger D-79112 Vacuum Pump N 035.1.2 AN.18).

Reactions were monitored by thin-layer chromatography (TLC) using aluminum-backed Merck 60 F_{254} silica gel plates and *n*-hexanes-ethyl acetate solvent systems. After visualization under ultraviolet light at 254 nm and 365 nm, the plates were developed by immersion in a solution containing a mixture of *p*-anisaldehyde (2.5%), acetic acid (1%), and sulfuric acid (3.4%) in 95% ethanol followed by heating. Solid compounds were mixed with SiO_2, redissolved in CH_2Cl_2, and concentrated under reduced pressure before purification through column chromatography using silica gel (Aldrich, 230-400 mesh) and EtOAc-hexanes mixtures. Compounds were systematically named following IUPAC recommendations with ChemDraw 20.0.0.41 (Perkin-Elmer, Waltham, MA, USA).

Abbreviations: bs = broad singlet; d = doublet; EtOAc = Ethyl acetate; EtOH = Ethanol; FT-MS = Fourier transform mass spectrometry; hep = heptet; MeOH = Methanol; NMR = Nuclear Magnetic Resonance; q = quintet; rt = room temperature; THF = Tetrahydrofuran; TLC = Thin Layer Chromatography; t = triplet.

1.2. Instruments and data analysis

1.2.1. NMR

NMR spectra were recorded in $CDCl_3$ or CD_3OD solutions on a Bruker NMR spectrometer (Bruker Advance III 400 MHz Ascend, 9.4 Tesla), and chemical shifts are reported on the δ scale (ppm) using the residual solvent signals [δ = 7.26 ppm (^{1}H, s, $CDCl_3$); δ = 77.0 ppm (^{13}C, t, $CDCl_3$)] and [δ = 4.870 ppm (^{1}H, s, Methanol-d_4), 3.31 ppm

(^{1}H, q, Methanol-d$_4$); δ = 49.0 ppm (^{13}C, hept, Methanol-d$_4$)] as internal standards. Coupling constants (*J*) are reported in Hz.

1.2.2. FT-MS

FT-MS analysis was done on a LTQ OrbitrapTM XL hybrid mass spectrometer (Thermo Fischer Scientific, Bremen, Germany) controlled by LTQ Tune Plus and Xcalibur 2.1.0.

1.3.Synthesis and characterization

1.3.1. 3,5-bis(4-Bromophenyl)pyrazin-2-amine

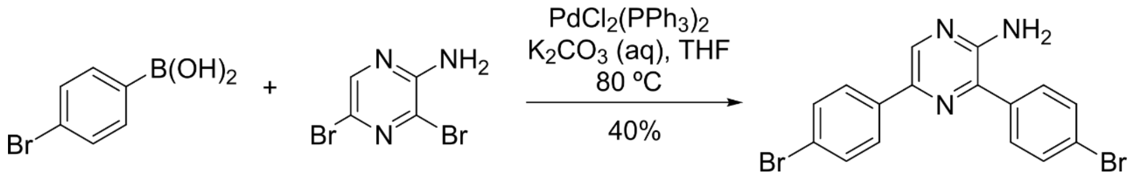

An aqueous solution of K_2CO_3 (1M, 9.25 mmol, 7.5 eq) was added to a solution of (4-bromophenyl)boronic acid (0.496 g, 2.469 mmol, 2 eq) and 3,5-dibromopyrazin-2-amine (0.312 g, 1.234 mmol, 1 eq) in THF (9 mL) and was deoxygenated with N_2. Then $PdCl_2(PPh_3)_2$ (0.043 g, 0.075 mmol, 0.05 eq) was added and the resulting mixture was stirred at 80 ºC until no starting material was detected by TLC (1:1 EtOAc-hexanes). The reaction mixture was cooled to rt and the aqueous phase discharged. The combined organic layers were washed with brine, dried with anhydrous sodium sulfate, filtered, and concentrated under reduced pressure to give an orange solid, which was purified by column chromatography (SiO_2, EtOAc/hexanes) to afford 3,5-bis(4-bromophenyl)pyrazin-2-amine as a yellow solid [0.200 g, R_f = 0.64 (10% EtOAc/hexanes)].

^{1}H NMR (400 MHz, $CDCl_3$) δ = 8.42 (s, 1H), 7.83 (d, *J* = 8.7, 2H), 7.71 (d, *J* = 8.7, 2H), 7.67 (d, *J* = 8.7, 2H), 7.57 (d, *J* = 8.7, 2H), 4.95 (bs, 2H). **^{13}C NMR** (101 MHz, $CDCl_3$) δ = 151 (C), 142.2 (C), 137.8 (CH), 132.4 (CH), 132.1 (CH), 130.1 (CH), 127.4 (CH), 123.7 (C), 122.7 (C).

1.3.2. 6,8-bis(4-Bromophenyl)-2-methylimidazo[1,2-a]pyrazin-3(7H)-one

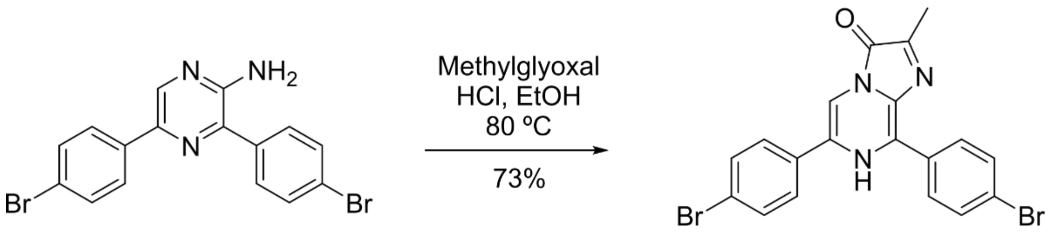

A solution of 3,5-bis(4-bromophenyl)pyrazin-2-amine (0.175 g, 0.432 mmol, 1 eq) and methylglyoxal (0.648 mmol, 1.5 eq) in EtOH (6 mL) was deoxygenated with N_2. Then the resulting mixture was cooled to 0 ºC, HCl (37%, 1.555 mmol, 3.6 eq) was added, the solution was stirred up to room temperature, and then stirred at 80 ºC until no starting material was detected by TLC (1:1 EtOAc-hexanes). The reaction mixture was cooled to room temperature and the resulting solution was concentrated under reduced pressure to give 6,8-bis(4-bromophenyl)-2-methylimidazo[1,2-*a*]pyrazin-3(7*H*)-one as an orange solid, which was redissolved in the minimum amount of EtOAc, precipitated with diethyl ether, and vacuum-dried.

^{1}H NMR (400 MHz, MeOD) δ = 8.85 (s, 1H), 8.12 (d, *J* = 8.7, 2H), 8.04 (d, *J* = 8.7, 2H), 7.89 (d, *J* = 8.6, 2H), 7.74 (d, *J* = 8.7, 2H), 2.59 (s, 3H). **FTMS-ESI (+)**: m/z: calcd for $[C_{19}H_{13}Br_2N_3O]^+$: 457.9425 $[M+H]^+$; found 457.9505 $[C_{19}H_{13}{}^{79}Br_2N_3O]^+$, 459.9482 $[C_{19}H_{13}{}^{79}Br^{81}BrN_3O]^+$, 461.9460 $[C_{19}H_{13}{}^{81}Br_2N_3O]^+$.

2. Supporting figures

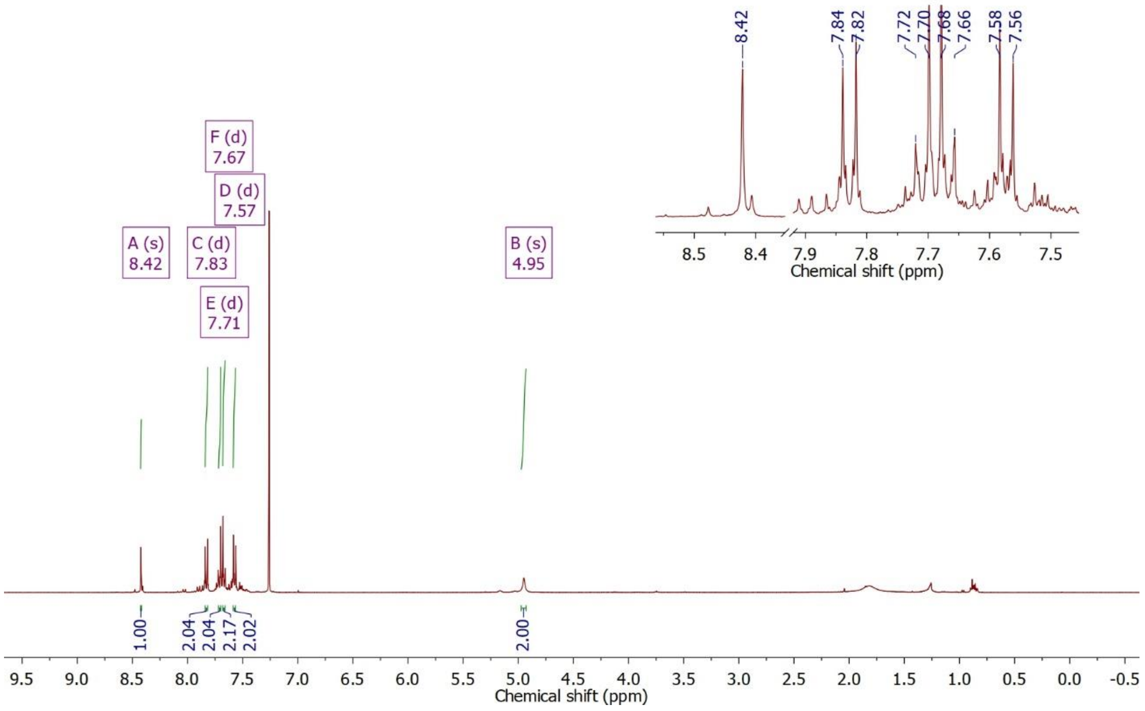

Figure S1. ^{1}H-NMR spectrum of 3,5-bis(4-bromophenyl)pyrazin-2-amine in $CDCl_3$. ^{1}H NMR (400 MHz, $CDCl_3$) δ = 8.42 (s, 1H), 7.83 (d, J = 8.7, 2H), 7.71 (d, J = 8.7, 2H), 7.67 (d, J = 8.7, 2H), 7.57 (d, J = 8.7, 2H), 4.95 (bs, 2H).

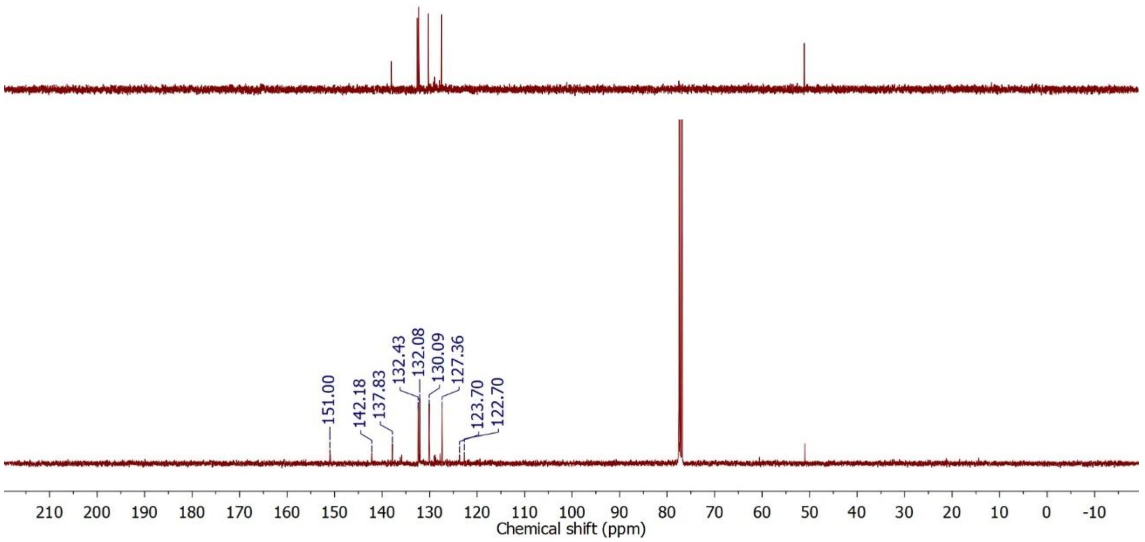

Figure S2. ^{13}C-NMR spectrum of 3,5-bis(4-bromophenyl)pyrazin-2-amine in $CDCl_3$. ^{13}C NMR (101 MHz, $CDCl_3$) δ = 151 (C), 142.2 (C), 137.8 (CH), 132.4 (CH), 132.1 (CH), 130.1 (CH), 127.4 (CH), 123.7 (C), 122.7 (C).

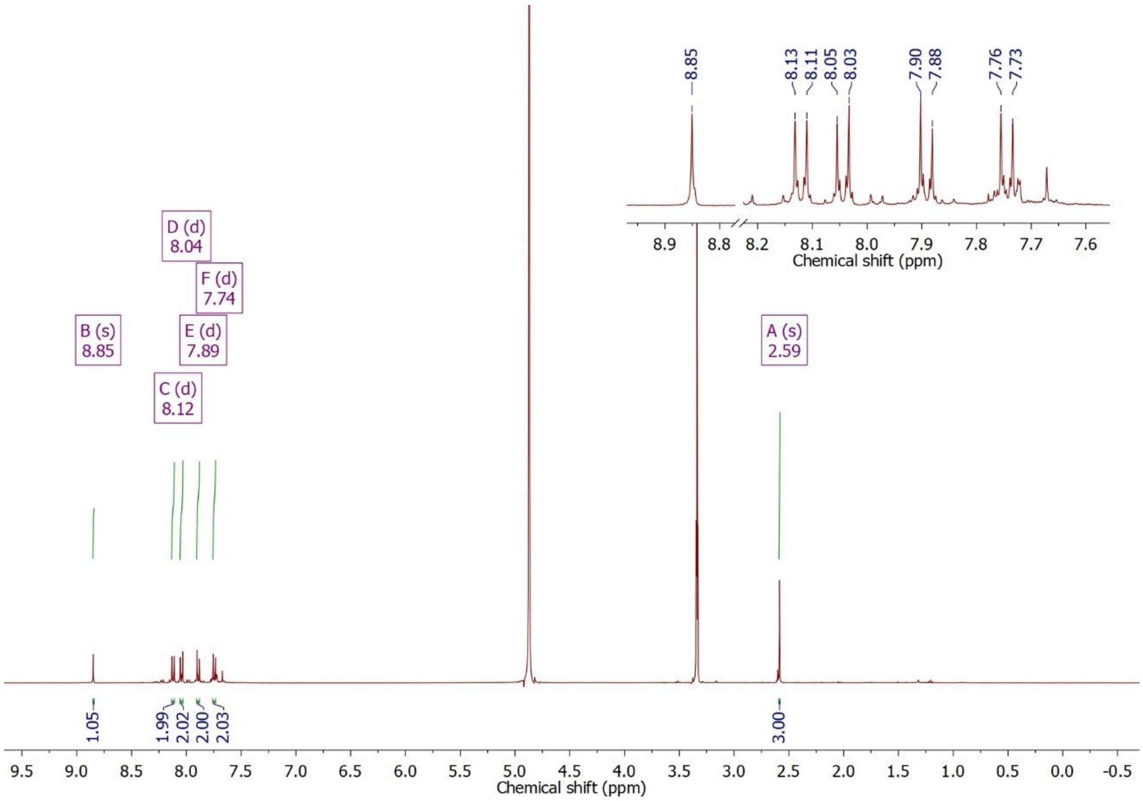

Figure S3. ^{1}H-NMR spectrum of 6,8-bis(4-bromophenyl)-2-methylimidazo[1,2-*a*]pyrazin-3(7*H*)-one in Methanol-d_4. ^{1}H NMR (400 MHz, MeOD) δ = 8.85 (s, 1H), 8.12 (d, *J* = 8.7, 2H), 8.04 (d, *J* = 8.7, 2H), 7.89 (d, *J* = 8.6, 2H), 7.74 (d, *J* = 8.6, 2H), 2.59 (s, 3H).

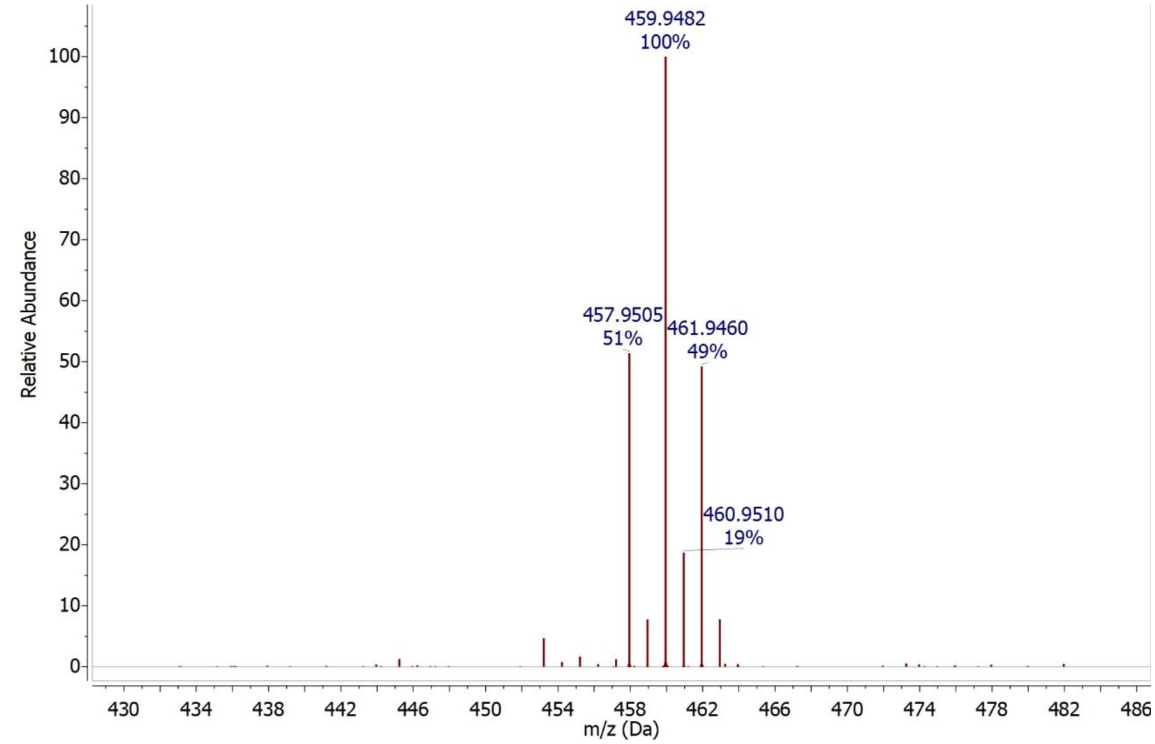

Figure S4. FT-MS spectrum of 6,8-bis(4-bromophenyl)-2-methylimidazo[1,2-*a*]pyrazin-3(7*H*)-one. FTMS-ESI (+): m/z: calcd for $[C_{19}H_{13}Br_2N_3O]^+$: 457.9425 $[M+H]^+$; found 457.9505 $[C_{19}H_{13}{}^{79}Br_2N_3O]^+$, 459.9482 $[C_{19}H_{13}{}^{79}Br^{81}BrN_3O]^+$, 461.9460 $[C_{19}H_{13}{}^{81}Br_2N_3O]^+$.
